# 

**Published:** 1807-01

**Authors:** 


### MEDICAL AND PHYSICAL

### JOURNAL.

THE

### MEDICAL AND PHYSICAL JOURNAL.

CONDUCTED BY

T. BRADLEY, M.D.

AND

R. BATTY, M.D.

VOL. XVII.

FROM JANUARY TO JUNE, 1807.

Surgeon Gen[illegible]
LIBRA[illegible]
5123
Washington, D.C.

*LONDON:*

PRINTED FOR RICHARD PHILLIPS, NO. 6, BRIDGE-STREET, BLACKFRIARS.

By William Thorne, Red Lion Court, Fleet Street.

### *TO CORRESPONDENTS.*

The Editors have received a communication from Mr. Wake, in which he states, that one of his children, who had been vaccinated in 1803, on being re-vaccinated about three weeks ago, had a "pustule, inflammation, and *discharge of matter*, (*which was large and copious*) all regularly appearing, proceeding and vanishing, exactly as before;" and hence concludes, that this last was a case of regular vaccine disease, occurring a second time on the same subject. We can assure him, that in the true vaccine disease there is no *discharge of matter* whatever, consequently not a large and copious one; therefore the last affection of the arm, was not a case of vaccination, but such as similar experiments have too often produced.

A constant reader will be particularly obliged to some of our correspondents, for information, through the medium of the Journal, respecting the manner of dissolving the elastic gum, (Caoutchouc) so as to render it applicable to form a coat on the flexible catheter, and for other surgical purposes.

---

Aminicus's Communication, relative to Ching's Lozenges, will appear in our next Number.

Practical Observations on Urinary Gravel and Stone; or, Diseases of the Bladder and Prostate Gland; and on Strictures of the Urethra. By Henry Johnson, 8vo. 5s. boards.

Observations on Indigestion; translated from the French Memoir of M. Daubenton. 8vo. 1s. 6d.

A Treatise on Insanity. By P. Pinel. 8vo. 9s. boards.

A Practical Treatise on the Powers of Cantharides when used Internally; demonstrated by Experiment and Observation. By J. Robertson, 8vo. 7s. boards.

Observations on Morbid Poisons; by Joseph Adams, M. D. F. L. S. in two Parts. 4to. 25s. boards.

Esculapius; or the Pocket Physician, a Collection of scarce aad curious Receipts in Medicine and Surgery. 2s. 6d.

---

### *Diseases and Casualties in London in the Year* 1806.

| | |
|---|---|
| Abortive and Stillborn | 956 |
| Abfcefs - - - | 93 |
| Aged - - - - - | 1380 |
| Ague - - - - - | 13 |
| Apoplexy & Suddenly | 348 |
| Afthma and Phthific | 382 |
| Bedridden - - - | 5 |
| Bile - - - - - - | 3 |
| Bleeding - - - - - | 24 |
| Burften and Rupture | 24 |
| Cancer - - - - - | 71 |
| Chicken Pox - - | 1 |
| Chin Cough - - - - | 0 |
| Childbed - - - - | 235 |
| Colds - - - - - | 12 |
| Colic, Gripes, &c. - | 21 |
| Confumption - - | 3996 |
| Convulfions - - | 3602 |
| Cough, and Hooping Cough - - - | 623 |
| Cow Pox - - - - - | 0 |
| Cramp - - - - - | 3 |
| Croup - - - - - | 44 |
| Diabetes - - - - - | 1 |
| Dropfy - - - - - | 763 |
| Eaten by Lice - - | 0 |
| Evil - - - - - - | 3 |
| Fevers of all kinds | 1354 |
| Fiftula - - - - - | 4 |
| Flux - - - - - - | 4 |
| French Pox - - - | 53 |
| Gout - - - - - | 101 |
| Gravel, Stone, and Strangury - - - | 27 |

| | |
|---|---|
| Grief - - - - - | 3 |
| Headmouldfhot, Horfe-fhoehead, and Water in the Head - | 199 |
| Jaundice - - - - - | 63 |
| Jaw Locked - - - | 2 |
| Impofthume - - - | 0 |
| Inflammation - - | 560 |
| Influenza - - - - - | 0 |
| Inoculation - - - | 0 |
| Itch - - - - - - | 1 |
| Lethargy - - - - - | 2 |
| Livergrown - - - | 13 |
| Lumbago - - - - - | 0 |
| Lunatick - - - - - | 146 |
| Meafles - - - - - | 530 |
| Mifcarriage - - - | 1 |
| Mortification - - | 285 |
| Palfy - - - - - | 138 |
| Palpitation of the Heart - - - - | 4 |
| Piles - - - - - - | 0 |
| Pleurify - - - - - | 24 |
| Purples - - - - | 2 |
| Quinfy - - - - - | 2 |
| Rafh - - - - - - - | 1 |
| Rheumatifm - - - | 6 |
| Scurvy - - - - - | 2 |
| Small Pox - - | 1158 |
| Sore Throat - - | 7 |
| Sores and Ulcers - - | 5 |
| St. Anthony's Fire - | 2 |
| Spafm - - - - - | 16 |
| Stoppage in the Stom. | 14 |

| | |
|---|---|
| St. Vitus's Dance - - | 0 |
| Surfeit - - - - - | [illegible] |
| Teeth - - - - - - | 481 |
| Thrufh - - - - - | 83 |
| Tumour - - - - - | 1 |
| Vomiting and Loofe-nefs - - - - - | 1 |
| Worms - - - - - | 6 |
| Broken Limbs - - | 3 |
| Broken Neck - - - | 0 |
| Bruifed - - - - - - | 2 |
| Burnt - - - - - | 36 |
| Choaked - - - - - | 0 |
| Drowned - - - | 132 |
| Exceffive Drinking - | 15 |
| Executed - - - - - | 4 |
| Found Dead - - - | 16 |
| Fractured - - - - - | 3 |
| Frozen - - - - - - | 0 |
| Killed by Falls and feveral other Accidents | 74 |
| Killed by Fighting - | 0 |
| Killed Themfelves - | 31 |
| Murdered - - - - - | 3 |
| Overlaid - - - - - | 0 |
| Poifoned - - - - - | 2 |
| Scalded - - - - - | 6 |
| Shot - - - - - - | 0 |
| Smothered - - - - | 2 |
| Starved - - - - - - | 1 |
| Strangled - - - - - | [illegible] |
| Suffocated - - - - - | 5 |

| | | | |
|---|---|---|---|
| Chriftened | Males - | 10452 | In all 20380 |
| | Females - | 9928 | |
| Buried - | Males - | 9215 | In all 17938 |
| | Females - | 8723 | |

Whereof have died,

| | | | |
|---|---|---|---|
| Under Two Years of Age - - | 5405 | Fifty and Sixty - - - - - | 1503 |
| Between Two and Five - - - | 2029 | Sixty and Seventy - - - - - | 1265 |
| Five and Ten - - - - - - | 822 | Seventy and Eighty - - - - | 859 |
| Ten and Twenty - - - - - - | 635 | Eighty and Ninety - - - - | 414 |
| Twenty and Thirty - - - - | 1329 | Ninety and a Hundred - - - | 99 |
| Thirty and Forty - - - - - - | 1782 | A Hundred - - - - - - - | 2 |
| Forty and Fifty - - - - - - | 1793 | A Hundred and Four - - - - | 1 |

Increafed in the Burials this Year 363.

